# Supplementary material for: Identification and functional analysis of non-coding regulatory small RNA FenSr3 in Bacillus amyloliquefaciens LPB-18
Source: PeerJ. 2023 May 15;11:e15236. doi: 10.7717/peerj.15236 (PMC10194069; doi:10.7717/peerj.15236)
Supplement: Supplemental Information 4 [file peerj-11-15236-s004.zip › KO/CK-vs-T1_map/map01200.html]

KEGG PATHWAY: Carbon metabolism - Reference pathway


|  |  |
| --- | --- |
| **Carbon metabolism - Reference pathway** |  |

[
Pathway menu
| Organism menu
| Pathway entry
|

Hide module list

| Show description
| User data mapping
|

Image (png) file

]

Carbon metabolism is the most basic aspect of life. This map presents an overall view of central carbon metabolism, where the number of carbons is shown for each compound denoted by a circle, excluding a cofactor (CoA, CoM, THF, or THMPT) that is replaced by an asterisk. The map contains carbon utilization pathways of glycolysis (map00010), pentose phosphate pathway (map00030), and citrate cycle (map00020), and six known carbon fixation pathways (map00710 and map00720) as well as some pathways of methane metabolism (map00680). The six carbon fixation pathways are: (1) reductive pentose phosphate cycle (Calvin cycle) in plants and cyanobacteria that perform oxygenic photosynthesis, (2) reductive citrate cycle in photosynthetic green sulfur bacteria and some chemolithoautotrophs, (3) 3-hydroxypropionate bi-cycle in photosynthetic green nonsulfur bacteria, two variants of 4-hydroxybutyrate pathways in Crenarchaeota called (4) hydroxypropionate-hydroxybutyrate cycle and (5) dicarboxylate-hydroxybutyrate cycle, and (6) reductive acetyl-CoA pathway in methanogenic bacteria.

|  |  |  |
| --- | --- | --- |
| Reference pathway | 184% 150% 122% 100% 82% 67% 55% | 图片下载 |

- **KEGG module**

- Energy metabolism
  - Carbon fixation
    - M00165 Reductive pentose phosphate cycle (Calvin cycle)- M00166 Reductive pentose phosphate cycle, ribulose-5P => glyceraldehyde-3P
      - M00167 Reductive pentose phosphate cycle, glyceraldehyde-3P => ribulose-5P
      - M00168 CAM (Crassulacean acid metabolism), dark
      - M00169 CAM (Crassulacean acid metabolism), light
      - M00172 C4-dicarboxylic acid cycle, NADP - malic enzyme type
      - M00171 C4-dicarboxylic acid cycle, NAD - malic enzyme type
      - M00170 C4-dicarboxylic acid cycle, phosphoenolpyruvate carboxykinase type
      - M00173 Reductive citrate cycle (Arnon-Buchanan cycle)
      - M00376 3-Hydroxypropionate bi-cycle
      - M00375 Hydroxypropionate-hydroxybutylate cycle
      - M00374 Dicarboxylate-hydroxybutyrate cycle
      - M00377 Reductive acetyl-CoA pathway (Wood-Ljungdahl pathway)
      - M00579 Phosphate acetyltransferase-acetate kinase pathway, acetyl-CoA => acetate
      - M00620 Incomplete reductive citrate cycle, acetyl-CoA => oxoglutarate
  - Methane metabolism
    - M00567 Methanogenesis, CO2 => methane- M00357 Methanogenesis, acetate => methane
      - M00356 Methanogenesis, methanol => methane
      - M00563 Methanogenesis, methylamine/dimethylamine/trimethylamine => methane
      - M00174 Methane oxidation, methanotroph, methane => formaldehyde
      - M00346 Formaldehyde assimilation, serine pathway
      - M00345 Formaldehyde assimilation, ribulose monophosphate pathway
      - M00344 Formaldehyde assimilation, xylulose monophosphate pathway
- Carbohydrate and lipid metabolism
  - Central carbohydrate metabolism
    - M00001 Glycolysis (Embden-Meyerhof pathway), glucose => pyruvate- M00002 Glycolysis, core module involving three-carbon compounds
      - M00307 Pyruvate oxidation, pyruvate => acetyl-CoA
      - M00009 Citrate cycle (TCA cycle, Krebs cycle)
      - M00010 Citrate cycle, first carbon oxidation, oxaloacetate => 2-oxoglutarate
      - M00011 Citrate cycle, second carbon oxidation, 2-oxoglutarate => oxaloacetate
      - M00004 Pentose phosphate pathway (Pentose phosphate cycle)
      - M00006 Pentose phosphate pathway, oxidative phase, glucose 6P => ribulose 5P
      - M00007 Pentose phosphate pathway, non-oxidative phase, fructose 6P => ribose 5P
      - M00580 Pentose phosphate pathway, archaea, fructose 6P => ribose 5P
      - M00005 PRPP biosynthesis, ribose 5P => PRPP
      - M00008 Entner-Doudoroff pathway, glucose-6P => glyceraldehyde-3P + pyruvate
      - M00308 Semi-phosphorylative Entner-Doudoroff pathway, gluconate => glycerate-3P
      - M00633 Semi-phosphorylative Entner-Doudoroff pathway, gluconate/galactonate => glycerate-3P
      - M00309 Non-phosphorylative Entner-Doudoroff pathway, gluconate/galactonate => glycerate
  - Other carbohydrate metabolism
    - M00012 Glyoxylate cycle- M00373 Ethylmalonyl pathway
      - M00740 Methylaspartate cycle
      - M00532 Photorespiration
      - M00013 Malonate semialdehyde pathway, propanoyl-CoA => acetyl-CoA
      - M00741 Propanoyl-CoA metabolism, propanoyl-CoA => succinyl-CoA
- Nucleotide and amino acid metabolism
  - Serine and threonine metabolism
    - M00020 Serine biosynthesis, glycerate-3P => serine
  - Cysteine and methionine metabolism
    - M00021 Cysteine biosynthesis, serine => cysteine

  

- **Reaction module**

- Carboxylic acid metabolism
  - Fatty acid synthesis and degradation
    - RM018 Beta oxidation in acyl-CoA degradation- RM020 Fatty acid synthesis using acetyl-CoA (reversal of RM018)
  - Acyl-CoA metabolism
    - RM019 Acyl-CoA conversion via dicarboxylate semialdehyde
